# Symbiotic NCR Peptide Fragments Affect the Viability, Morphology and Biofilm Formation of *Candida* Species

**DOI:** 10.3390/ijms22073666

**Published:** 2021-04-01

**Authors:** Bettina Szerencsés, Attila Gácser, Gabriella Endre, Ildikó Domonkos, Hilda Tiricz, Csaba Vágvölgyi, János Szolomajer, Dian H. O. Howan, Gábor K. Tóth, Ilona Pfeiffer, Éva Kondorosi

**Affiliations:** 1 Department of Microbiology, Faculty of Science and Informatics, University of Szeged, H-6726 Szeged, Hungary; betti414@gmail.com (B.S.); gacsera@bio.u-szeged.hu (A.G.); csaba@bio.u-szeged.hu (C.V.); 2 Biological Research Centre, Institute of Plant Biology, H-6726 Szeged, Hungary; endre.gabriella@brc.hu (G.E.); domonkos.ildiko@brc.hu (I.D.); tiriczh@brc.hu (H.T.); 3 Department of Medical Chemistry, University of Szeged, H-6720 Szeged, Hungary; szolomajer.janos@med.u-szeged.hu (J.S.); dian.howan@med.u-szeged.hu (D.H.O.H.); toth.gabor@med.u-szeged.hu (G.K.T.); 4 MTA-SZTE Biomimetic Systems Research Group, University of Szeged, H-6720 Szeged, Hungary

**Keywords:** NCR peptide, antifungal activity, *Candida*, morphological switch, biofilm

## Abstract

The increasing rate of fungal infections causes global problems not only in human healthcare but agriculture as well. To combat fungal pathogens limited numbers of antifungal agents are available therefore alternative drugs are needed. Antimicrobial peptides are potent candidates because of their broad activity spectrum and their diverse mode of actions. The model legume *Medicago truncatula* produces >700 nodule specific cysteine-rich (NCR) peptides in symbiosis and many of them have in vitro antimicrobial activities without considerable toxicity on human cells. In this work we demonstrate the anticandidal activity of the NCR335 and NCR169 peptide derivatives against five *Candida* species by using the micro-dilution method, measuring inhibition of biofilm formation with the XTT (2,3-Bis-(2-Methoxy-4-Nitro-5-Sulfophenyl)-2H-Tetrazolium-5-Carboxanilide) assay, and assessing the morphological change of dimorphic *Candida* species by microscopy. We show that both the N- and C-terminal regions of NCR335 possess anticandidal activity as well as the C-terminal sequence of NCR169. The active peptides inhibit biofilm formation and the yeast-hypha transformation. Combined treatment of *C. auris* with peptides and fluconazole revealed synergistic interactions and reduced 2-8-fold the minimal inhibitory concentrations. Our results demonstrate that shortening NCR peptides can even enhance and broaden their anticandidal activity and therapeutic potential.

## 1. Introduction

Fungal infections are increasing and represent serious health threat particularly in the increasing population of immunosuppressed patients. Invasive fungal infections are generally associated with high mortality [1] and the use of effective antifungal drugs is crucial for the outcome of the patient’s disease. Unfortunately, the repertoire of antifungal agents is rather limited and for systemic therapy only three classes of drugs are available: polyenes, triazoles and echinocandins. In addition to the limited spectrum of antifungals, similarly to bacterial resistance against the antibiotics, development of drug resistance against these agents has been reported in *Candida* and *Cryptococcus* species and in some moulds [2]. The problem to fight fungal infections also exists in agriculture leading to significant loss in agricultural productivity. Therefore, there is a great and urgent need for novel types of antifungal agents, and alternative antifungal strategies both in healthcare therapies and in the agriculture [3].

Antimicrobial peptides (AMPs) have a great potential as an untapped source of novel biologically active agents to fight against bacteria, fungi, parasites, and viruses. AMPs are produced in all domains of life and are part of the host innate immune response protecting the host against microbial infections. AMPs show extraordinary diversity in nature, their length varies between 10 and 60 amino acids and almost all AMPs are cationic. Most prevalently AMPs act directly on the microbial membranes leading to the loss of membrane potential, leakage and finally death of cells. However, a remarkable quality of many AMPs is that they act by multiple mechanisms and can have numerous intracellular biological targets that are distinct from targets of the traditional antibiotics/drugs. The antimicrobial peptide database lists 3081 peptides mainly from natural sources with an average peptide length of 33 amino acids and a net charge of +3.3 [4]. Plant AMPs are poorly represented in the AMP databases, although even a single plant can produce close to 1000 different AMPs. Defensins, secreted peptides having 8 or 10 conserved cysteine residues, represent the major class of AMPs in plants. In addition, certain legumes, belonging to the Inverted Repeat Lacking Clade (IRLC) such as alfalfa and other *Medicago* species or pea, clover, lentil, vetch, and other plants in this clade, have evolved a defensin-related but unique gene family which codes for nodule-specific cysteine rich (NCR) peptides produced only in *Rhizobium*-legume symbiosis. *Medicago truncatula* forms nitrogen fixing symbiosis with *Sinorhizobium meliloti* soil bacteria. The interaction between the host plant and the bacterium leads to the formation of a symbiotic organ, the root nodule where the bacteria live inside the nodule cells. In these infected cells the plant manipulates the fate of bacteria, by transforming them to non-cultivable, huge polyploid bacteria with the help of NCR peptides [5,6]. In *Medicago truncatula,* more than 700 genes code for NCR peptides [7,8]. NCRs have 4 or 6 cysteines in conserved position and otherwise high divergence in their amino acid composition and sequence resulting in a great diversity in their physicochemical properties that is also reflected by the wide range of isoelectric point (pI) of peptides from 3.2 to 11.2. Many of these symbiotic NCR peptides have in vitro strong antimicrobial activities [9]. Cationic NCRs such as NCR247 (pI: 10.15) and NCR335 (pI: 11.22) show broad range and partially overlapping activities, indicating that in addition to the positive net charge, the amino acid sequence contributes to the antimicrobial properties [10]. NCR247 acts via multiple mechanisms, interacting with the bacterial membranes and by entering the cells with many bacterial proteins and affecting both transcription and translation and inhibiting cell division by interacting with the conserved bacterial FtsZ protein required for septum formation and cell division [11,12]. Shorter chimeric derivatives of NCR247 gained even higher activities and were also able to kill the most resistant and problematic ESKAPE bacteria at low concentrations making them comparable or even superior to third generation antibiotics with the advantage of lack of cytotoxicity on human cells [13].

In addition to bacteria, NCRs can also kill fungi. Previously we investigated the antifungal effect of 19 NCR peptides with pI from 3.6 to 11.2 against *Candida albicans* and found that peptides with pI > 9.5 inhibited the growth of *C. albicans* [14]. The minimal inhibitory concentrations (MIC) of these cationic NCR peptides (NCR192, NCR137, NCR147, NCR 280, NCR183, NCR247, NCR044, NCR030, NCR335) were in the range of 1.4–10 µM. For example, the MIC of NCR335 (pI: 11.22) and NCR044 (pI: 10.32) was 1.4–2.5 µM. Importantly, these concentrations of cationic NCR peptides, which efficiently eliminated *C. albicans* did not affect the viability of vaginal epithelial cells while they inhibited the *C. albicans*-induced killing of epithelial cells. NCR044 exhibited potent antifungal activity against plant fungal pathogens as well, such as various *Fusarium* species and *Botrytis cinerea* and was shown to act by multifaceted mechanisms [15]. Thus, cationic NCRs, due to their potent antifungal activity, negligible host toxicity and low resistance rates emerge as potential candidates for developing new antifungal therapies.

Weakly cationic peptides such as NCR169 (pI: 8.45) or neutral and anionic NCRs were inactive against *C. albicans* in the studied concentration range [14]. However, we noticed that, unlike the full sequence of NCR169, its C-terminal half was highly cationic (pI: 10.1) leading us to assume that this part may have antimicrobial activity.

In this study, the growth inhibitory and fungicide effects of eleven NCR335 derivatives, and the C-terminal region of NCR169 were tested against 7 strains of 5 human pathogenic *Candida* species. We also investigated how the most effective peptides act on the biofilm formation and on the yeast to hypha morphological switch of *C. albicans* and *C. tropicalis* as hyphal growth represents an important virulence factor in these dimorphic species [16,17]. Moreover, synergism was demonstrated between peptides and between peptides and the antifungal drug, fluconazole against *C. auris* which significantly reduced the MIC values and helps to overcome the mild toxicity of these peptides and antifungal drugs.

## 2. Results

### 2.1. Design and Synthesis of NCR335 and NCR169 Peptide Derivatives

NCR335 is not only one of the most cationic NCR peptides but it is exceptional due to its 64 amino acid (RLNTTFRPLNFKMLRFWGQNRNIMKHRGQKVHFSLILSDCKTNKDCPKLRRANVRCRKSYCVPI) long sequence compared to the average length of 33 amino acids of NCRs. NCR335 is composed of an NCR-specific sequence with four conserved cysteine residues on the C-terminal 33 amino acid long sequence. The 31 amino acid long N-terminal sequence (RLNTTFRPLNFKMLRFWGQNRNIMKHRGQKV) is unrelated to the NCR sequences and lacks cysteine residues but has +8.1 net charge and pI: 12.72, which raised the possibility that this sequence may also have antimicrobial activity. Therefore, we investigated separately the activity of the N- and C-terminal regions of NCR335. To identify the regions responsible for antifungal activities peptide fragments were synthetized from the N-terminal sequence corresponding to the 1–19, 1–15, 7–21, 16–29 amino acid sequences, while from the 33 amino acid long NCR335C peptide the 13–33, 17–33, 17–27, 1–8, 9–16, 1–16 sequences were synthetized (Table 1). Except for NCR335C_1–8_ and NCR335C_9–16_, all peptides have significant + net charge and high pI.

In the case of NCR169 (EDIGHIKYCGIVDDCYKSKKPLFKIWKCVENVCVLWYK), we investigated the C-terminal 22 amino acid long sequence (KSKKPLFKIWKCVENVCVLWYK), NCR169C_17–38_, with a net charge of +5.9 and a pI of 10.48 in linear and a net charge of +6 and a pI of 11.01 in oxidized form, and by replacing the two tryptophan residues (W_10_ and W_20_) with alanine (Table 1). This latter substitution was made because tryptophan residues could be required for interaction with microbial membranes and can play a critical role in the antimicrobial activity [18].

**Figure A1:**
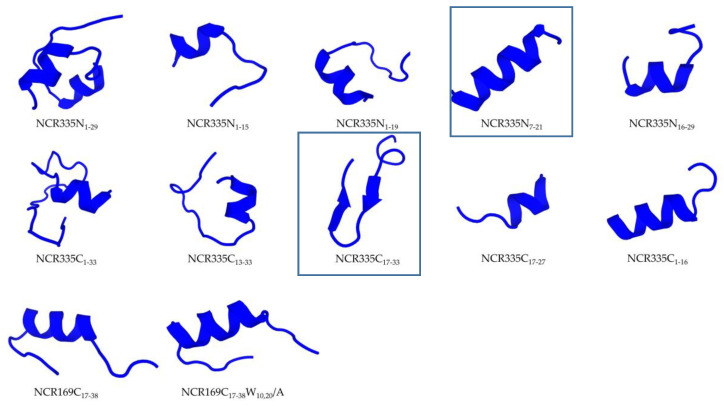
Predicted structure of the NCR peptide derivatives. Folding of the active NCR335N_7–21_ and NCR335C_17–33_ peptides is highlighted in the frames.

The 3D folding of peptides longer than 8 amino acids was predicted with the PEP-FOLD3 tool (Figure A1). The folding of NCR335N_1–29_ indicates a relatively disordered structure, with a relatively short alpha helical segment in the N- and C-terminal half of the peptide. The two halves of the molecule (NCR335N_1–19_ and NCR335N_16–29_) retain the original steric structure, a short alpha helical region on each fragment. Interestingly the predicted 3D structure of NCR335N_7–21_ appears to be almost exclusively alpha helical and different from the other NCR335N derivatives. In NCR335C_1–33_ a helical segment is predicted at the N terminus in the otherwise disordered structure. Similar folding is predicted for NCR335C_17–27_ and NCR335C_13–33_. In contrast, the 3D structure of NCR335C_17–33_ appears to be dramatically different: the helical part is absent while two short antiparallel β strands are formed. In NCR169C_17–38_, a helical region is predicted in the middle, while the ends are disordered. Replacement of tryptophan residues to alanine does not change the 3D structure.

### 2.2. NCR Based Peptides Affect the Growth and Survival of Candida Strains

The antifungal activity of all the 14 peptides was tested against the following opportunistic human pathogen *Candida* species and strains: *C. albicans* strains ATCC 10231, SC 5314 and SZMC 1458, *C. auris* 0381, *C. glabrata* CBS 138, *C. parapsilosis* CBS 604 and *C. tropicalis* CBS 94. To determine the minimal inhibitory concentration of the peptides, the strains were incubated with two-fold serial dilutions of the peptides from 25 μM to 1.56 μM (Table 2).

Both the N- and the C-terminal regions of NCR335 exhibited anti-*Candida* activity. Of the NCR335N peptides, NCR335N_7–21_ displayed the broadest activity; except *C. parapsilosis* and *C. auris.* It inhibited the growth of *C. albicans* strains ATCC 10231, SC 5314 and SZMC 1458 and *C. glabrata* CBS 138 at 25 µM and *C. tropicalis* CBS 94 at 6.25 µM. Compared to NCR335N_7–21_, the overlapping NCR335N_1–19_ peptide, which lacks the last two amino acids, and has an N-terminal extension of seven amino acids, remained active only against *C. albicans* ATCC 10231 (MIC 25 µM), but became effective against *C. parapsilosis* (MIC 12.50 µM). NCR335N_1–15_ and NCR335N_16–29_ did not affect the growth of the studied strains in this concentration range.

The C-terminal part of NCR335, NCR 335C_1–33_, with the characteristic pattern of cysteine residues, was effective against all strains except *C. auris* and *C. tropicalis* with MICs of 12.50 and 25 µM. The shorter peptide, NCR335C_17–33_ corresponding to the last 17 amino acids of NCR 335C_1–33_ showed even higher activity and became remarkably effective against *C. tropicalis* (MIC 3.12 µM). The four amino acid longer NCR335C_13–33_ peptide was only effective against *C. albicans* SC 5314. NCR335C_17–27_, NCR335C_1–8_, NCR335C_9–16_ and NCR335C_1–16_ were ineffective, having no effect on the growth of any of the tested strains.

The NCR169 derivatives NCR169C_17–38_ and NCR169C_17–38_ox proved to be active against all the examined strains including *C. auris* but were ineffective against *C. parapsilosis.* The MIC values of both peptides ranged from 3.12 µM to 25 µM, and the oxidized form NCR169C_17–38_ox was more efficient against the *C. albicans* strains and *C. glabrata*. NCR169C_17–38_W_10,20_/A lost the activity against *C. auris* but maintained activity against the other strains.

The effectiveness of these peptides was compared to that of two antifungal agents: fluconazole and amphotericin B (Table 2). Fluconazole is used to treat various fungal and yeast infections including candidiasis. Fluconazole was tested in a concentration range from 200 to 0.78 µM and was highly effective against *C. albicans* SZMC 1458 (MIC 1.56 µM), followed by *C. parapsilosis* (MIC 6.25 µM), *C. albicans* SC 5314 (MIC 12.50 µM), *C. auris* (MIC 25 µM), *C. tropicalis* (MIC 100 µM), *C. glabrata* (MIC 200 µM) and *C. albicans* ATCC 10231 (MIC 200 µM). The MIC values of amphotericin B were between 1.56 and 6.25 µM: *C. glabrata* was the most sensitive (MIC 1.56 µM), followed by the three *C. albicans* strains and *C. parapsilosis* (MIC 3.12 µM) and then by *C. auris* (MIC 6.25 µM). While amphotericin B seems to be highly effective, it is only used for life-threatening fungal infections due to its serious side effects.

**Figure A2:**
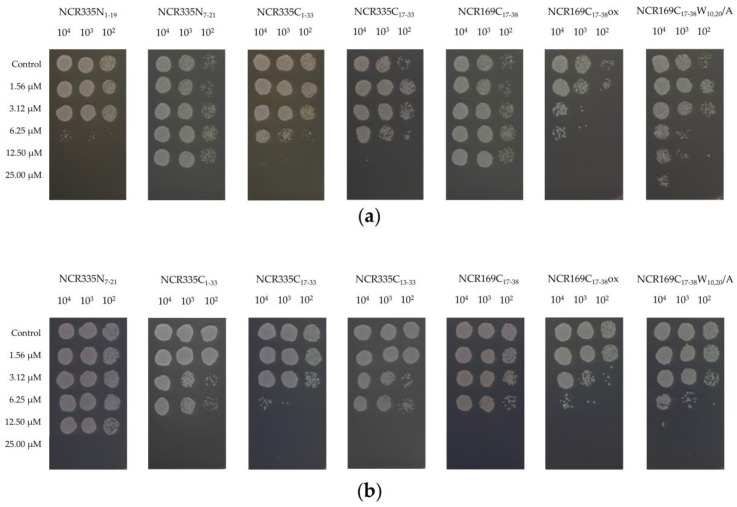

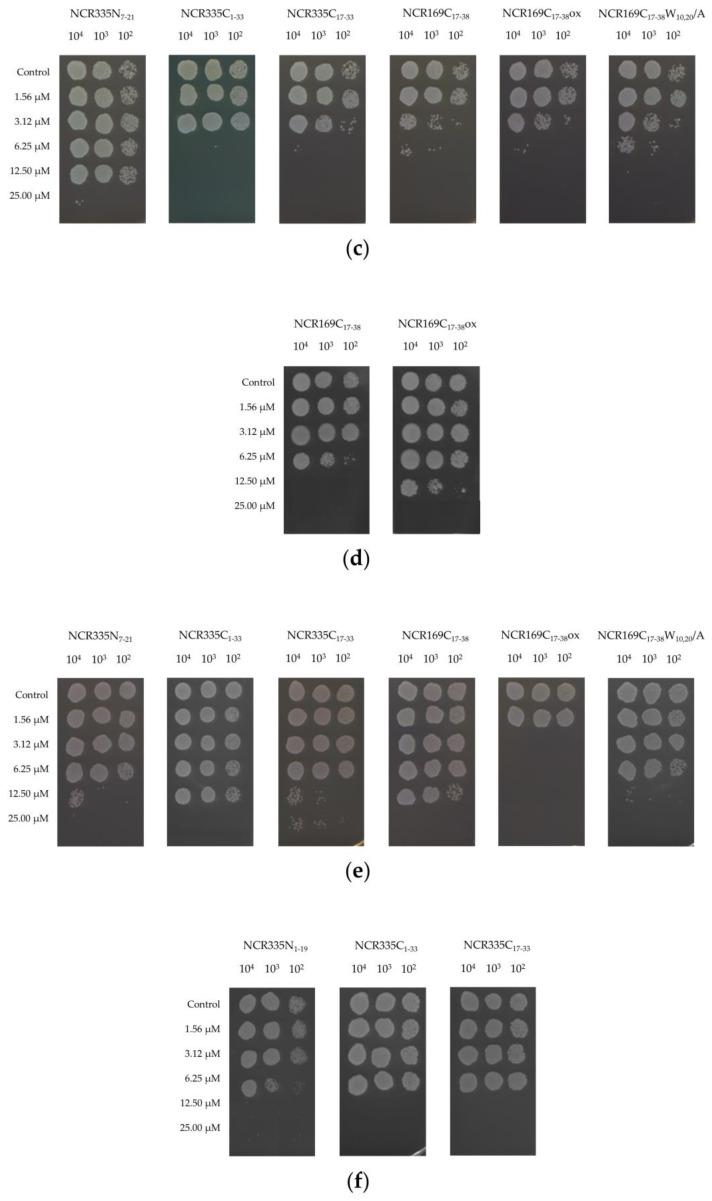

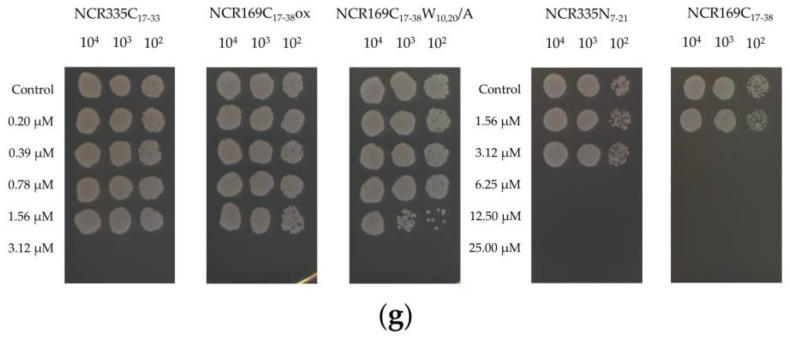
Growth of *Candida* species after AMP-treatment. (**a**) *Candida albicans* ATCC 10231, (**b**) *C. albicans* SC 5314, (**c**) *C. albicans* SZMC 1458, (**d**) *C. auris* 0381, (**e**) *C. glabrata* CBS 138, (**f**) *C. parapsilosis* CBS 604, (**g**) *C. tropicalis* CBS 94. Peptides and their applied concentration are indicated in the panels.

To determine whether the inhibitory effect of the peptides is generated by fungistatic or fungicide action, the *Candida* cells were treated with the peptides in the same concentration range (1.56–25 µM) as in the growth inhibition assay. After 24 h 5 µL sample from the untreated control cells and from the peptide treated cells was added to 95 µL water and diluted further 10- and 100-fold. From each dilution, 5 µL was placed on solid medium and the growth of the control and peptide treated cells was compared after 48 h incubation at 30 °C. This assay confirmed that all the effective peptides, NCR335N_1–19_, NCR335N_7–21_, NCR335C_1–33_, NCR335C_13–33,_ NCR335C_17–33,_ NCR169C_17–38_, NCR169C_17–38_ox, NCR169C_17–38_W_10,20_/A provoked growth inhibition through fungicidal actions (Figure A2). The fungicide concentrations, required for the complete elimination of the *Candida* cells, were either equal to or twice higher than the minimum inhibitory concentrations presented in Table 2.

### 2.3. NCR335C_17–33_ and NCR169C_17–38_ Reduce Hyphae Formation

**Figure 1:**
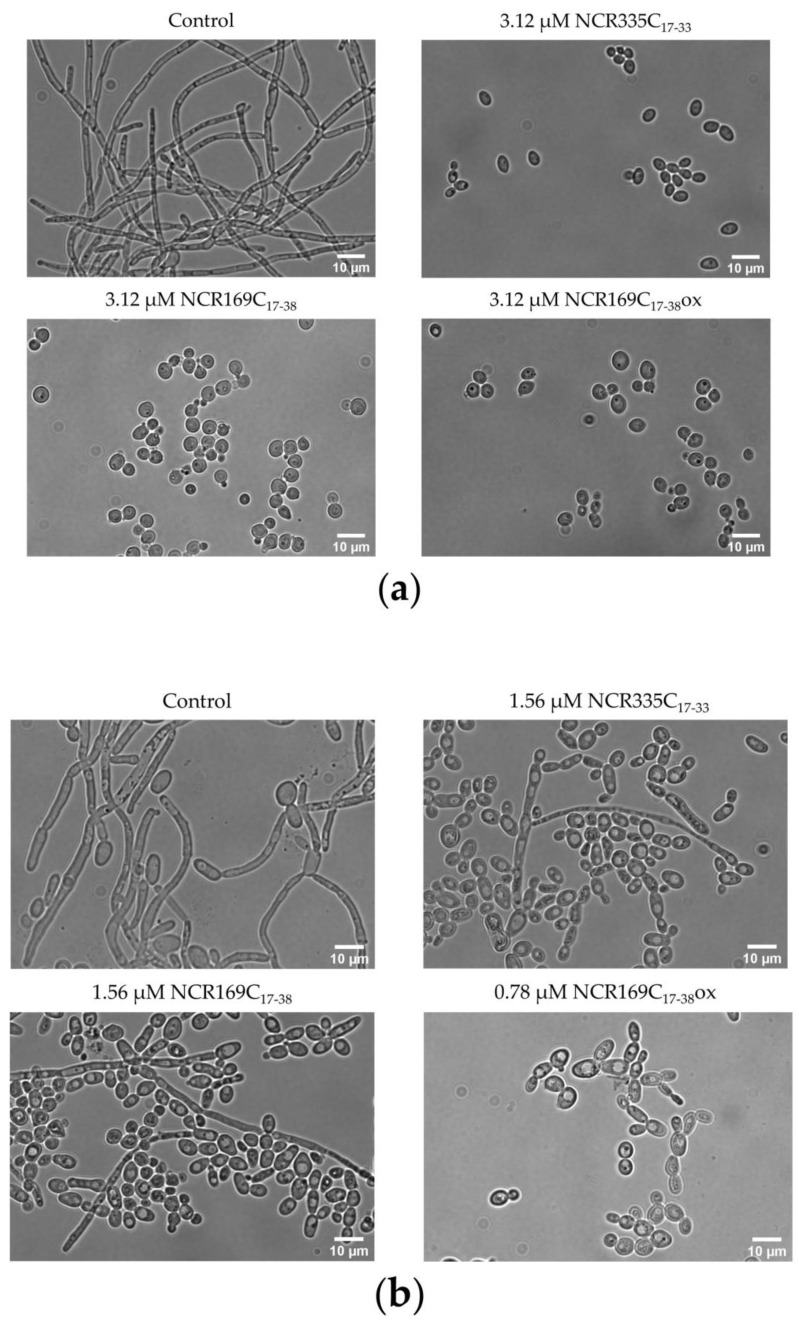
Morphology of the untreated control and the NCR335C_17–33_-, NCR169C_17–38_- or NCR169C_17–38ox_-treated *C. albicans* ATCC 10231 (**a**) and *C. tropicalis* CBS 94 (**b**) cells. Scale bars represent 10 µm.

As hyphae formation is an important virulence factor, we investigated how the broad range activity peptides, NCR335C_17–33_, NCR169C_17–38_ and NCR169C_17–38_ox affect the morphological switch of *C. albicans* ATCC 10231 and *C. tropicalis* between the hyphal and yeast growth forms at sub-lethal concentrations (Figure 1). Observing the cells after 48 h of cultivation with bright field microscopy revealed that treatment of *C. albicans* ATCC 10231 with 3.12 μM peptides completely inhibited the hyphal growth, which was the characteristic growth form of the control culture. In the untreated control culture of *C. tropicalis* the hyphal growth was predominant but shifted to the yeast form in the cultures treated with NCR335C_17–33_ and NCR169C_17–38_ at 1.56 μM and was the only form of the culture treated with NCR169C_17–38_ox at 0.78 μM. However, not only the morphology changed, but the cell number also declined, particularly in the latter case.

### 2.4. The Anticandidal NCR Peptide Derivatives Inhibit Biofilm Formation

**Figure 2:**
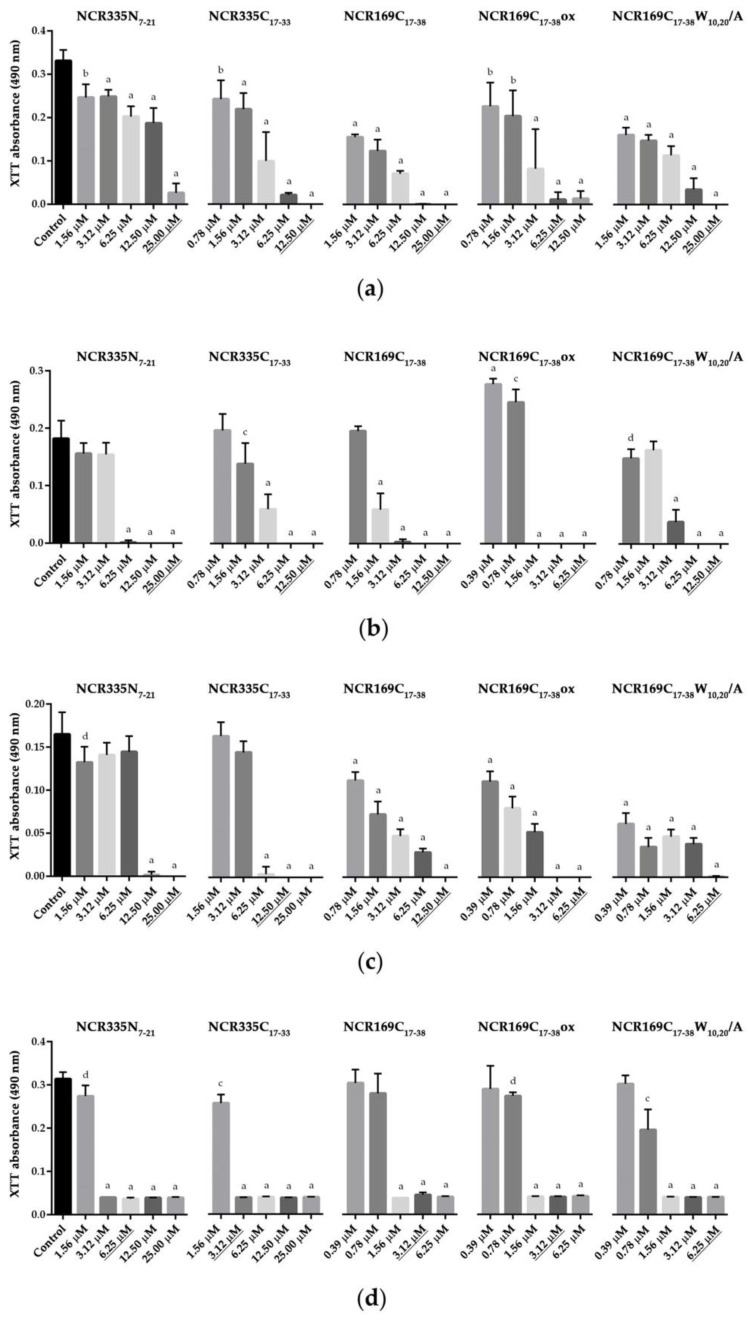
Effect of the peptides on biofilm formation of *C. albicans* and *C. tropicalis*. *C. albicans* ATCC 10231 (**a**); *C. albicans* SC 5314 (**b**); *C. albicans* SZMC 1458 (**c**) and *C. tropicalis* CBS 94 (**d**). Underlined concentrations correspond to the minimal inhibitory concentration (MIC) of a given peptide. The XTT absorbance values represent the mean ± standard deviation calculated from three independent experiments (a, *p* ≤ 0.0001, b, *p* ≤ 0.001, c, *p* ≤ 0.01, d, *p* ≤ 0.05, unpaired *t* test).

Biofilm formation is another important virulence factor for *C. albicans* and *C. tropicalis*, as cells located in the biofilm are more resistant to the antifungal agents due to the encompassing matrix that protects against the penetration of the antifungal drugs. Consequently, biofilm is a major source of persistent or recurrent infections [19]. The biofilm inhibitory ability of NCR335N_7–21_, NCR335C_17–33_, NCR169C_17–38_, NCR169C_17–38_ox and NCR169C_17–38_W_10,20_/A was tested on the three strains of *C. albicans* and *C. tropicalis,* by cultivating the strains in the presence of increasing concentrations of peptides for 72 h (Figure 2). The viability of the biofilm located cells was measured by the XTT (2,3-Bis-(2-Methoxy-4-Nitro-5-Sulfophenyl)-2H-Tetrazolium-5-Carboxanilide) assay. All five peptides significantly inhibited biofilm formation at the MICs or lower concentrations (*p* ≤ 0.0001). However, the *Candida* strains exhibited certain differences in their sensitivity to the peptides. NCR335N_7–21_ was effective at MIC in *C*. *albicans* ATCC 10231, at half MIC in *C. albicans* SZMC 1458 and *C. tropicalis* and at one-fourth of the MIC in *C. albicans* SC 5314. NCR335C_17–33_ acted at the MIC in *C. tropicalis* and at half MIC in the other strains. NCR169C_17–38_ was required at MIC in *C. albicans* SZMC 1458, at half MIC in *C*. *albicans* ATCC 10231 and *C. tropicalis* and at one-fourth of the MIC in *C. albicans* SC 5314. NCR169C_17–38_ox acted at the MIC in *C*. *albicans* ATCC 10231, at half MIC in *C. albicans* SZMC 1458 and *C. tropicalis* and at one-fourth of the MIC in *C. albicans* SC 5314. NCR169C_17–38_W_10,20_/A was required at MIC in *C. albicans* SZMC 1458, at half MIC in *C. albicans* ATCC 10231 and *C. albicans* SC 5314 and at one-fourth of the MIC in *C. tropicalis*. Thus, biofilm inhibition was the most effectively achieved by NCR335N_7–21_, NCR169C_17–38_ and NCR169C_17–38_ox in *C. albicans* SC 5314 and by NCR169C_17–38_W_10,20_/A in *C. tropicalis.*

### 2.5. NCR335C_17–33_ and NCR169C_17–38_ Drastically Reduce the Hyphal Form in Biofilms

**Figure 3:**
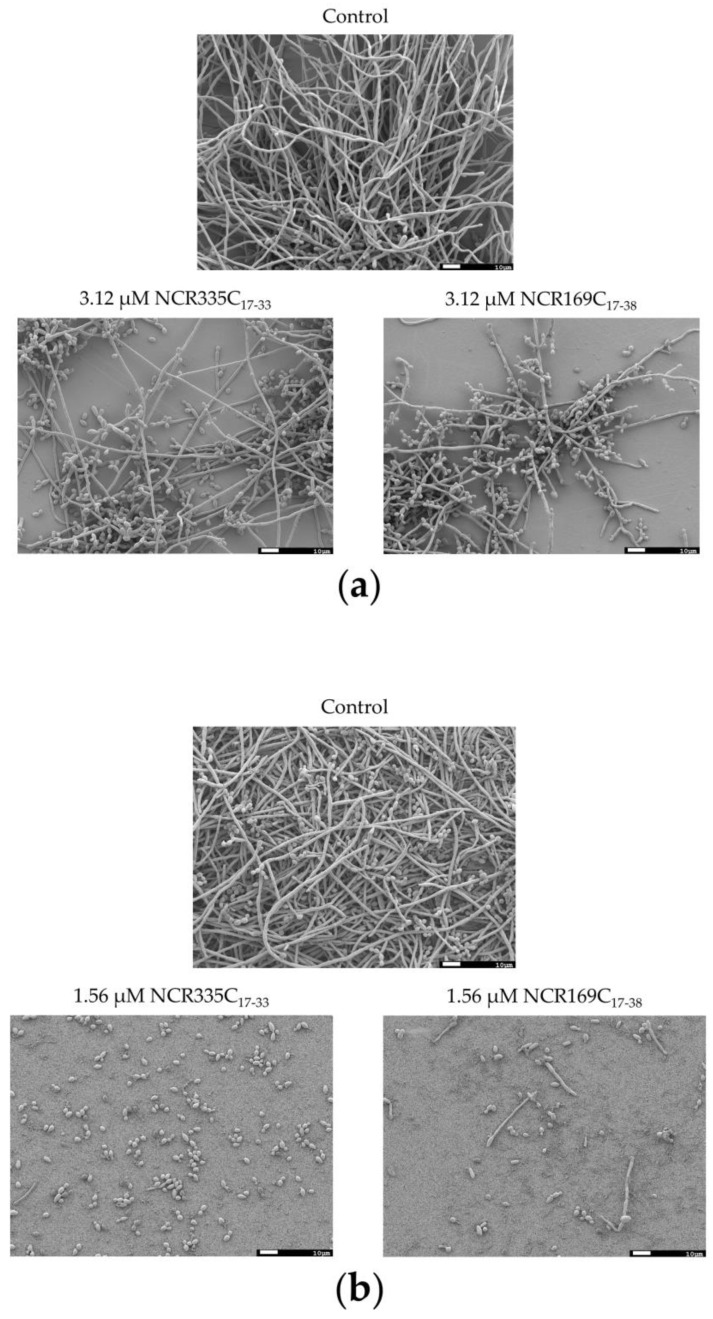
Morphology of the biofilm-located *C. albicans* ATCC 10231 (**a**) and *C. tropicalis* CBS 94 (**b**). The strains were cultivated without (control) or with NCR335C_17–33_ or NCR169C_17–38_ for 72 h and the cells were visualized by scanning electron microscopy. Scale bars represent 10 µm.

*Candida* biofilms are complex structures that may comprise different cell types [20], therefore the morphology of the cells was also investigated in the biofilms of *C. albicans* ATCC 10231 and *C. tropicalis* after the treatment with NCR335C_17–33_ and NCR169C_17–38_ at sub-lethal concentrations. Both peptides caused significant biofilm inhibition in *C. albicans* ATCC 10231 and *C. tropicalis* (Figure 2). Accordingly, the scanning electron microscopy (SEM) images showed lower structural complexity of the biofilms in both species after the treatment with NCR335C_17–33_ or NCR169C_17–38_ compared to the control biofilms (Figure 3). Both peptides reduced the abundance of the hyphal form and fostered more the yeast form resulting in decreased ratios of the hyphae/yeast form. *C. tropicalis* was particularly sensitive to the peptides; at 1.56 μM NCR335C_17–33_ practically only the yeast form was present, while at 1.56 μM NCR169C_17–38_ the cell number was drastically reduced, and the yeast form was also dominant in the biofilm.

### 2.6. Combined Treatment of C. auris with NCR Peptide Derivatives and Fluconazole Reveals Synergism

In therapy, application of two or more antibiotics simultaneously can be more effective than each alone. *C. auris* is one of the least treatable *Candida* species, and in this study only NCR169C_17–38_ (MIC 6.25 µM) and NCR169C_17–38_ox (MIC 12.50 µM) were active against this species as well as fluconazole (MIC 25 µM) and amphotericin B (MIC 6.25 µM). Therefore, we investigated whether combined application of these peptides with fluconazole or NCR335C_17–33_, which was active against other *Candida* species but not against *C. auris*, could reduce MIC values and reveal synergism by checkerboard titration assay (Table 3). Amphotericin B, due to its high toxicity was not included in this assay. Synergism was found in three combinations: (i) fluconazole and NCR169C_17–38_ox, (ii) NCR169C_17–38_ and NCR169C_17–38_ox and (iii) NCR169C_17–38_ and NCR335C_17–33_. In these cases, the growth inhibition was achieved when 6.25 µM fluconazole was combined with 1.56 µM NCR169C_17–38ox_, 3.12 µM NCR169C_17–38_ with 1.56 µM NCR169C_17–38_ox or 1.56 µM NCR169C_17–38_ with 3.12 µM NCR335C_17–33_. In the case of fluconazole (12.50 µM) and NCR169C_17–38_ (0.78 µM) additivity was observed. The action of fluconazole and NCR335C_17–33_ was indifferent.

### 2.7. Anti-Candida NCR Peptides Exhibit No or Only Moderate Cytotoxicity on Human Keratinocytes

**Figure A3:**
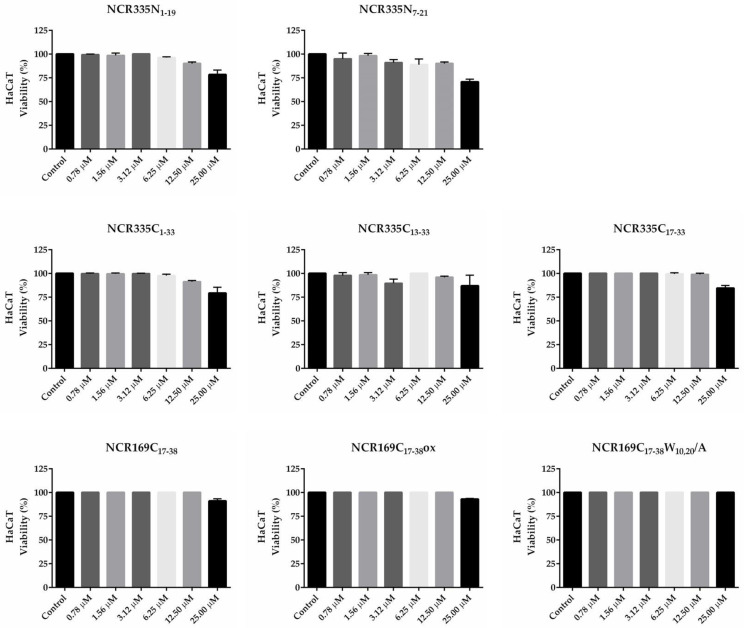
Cytotoxicity of the peptides on HaCaT cells. The viability of human keratinocytes was checked by MTT assay after 48 h of peptide treatment.

Cytotoxicity of antifungal agents, such as amphotericin B, may limit or hamper their therapeutic use. The *Medicago* NCR peptides tested to date did not show any or only minor toxicity, however, we could not exclude the possibility that the increased antifungal activity of these truncated NCR derivatives provokes cytotoxicity. Thus, human keratinocyte HaCaT cells were treated with NCR335N_1–19_, NCR335N_7–21_, NCR 335C_1–33_, NCR335C_13–33_, NCR169C_17–38_, NCR169C_17–38_ox and NCR169C_17–38_W_10,20_/A in a concentration range from 0.78 to 25 µM for 48 hrs and the viability of cells was determined by the MTT assay and compared to the viability of the control cultures (Figure A3). None of the peptides displayed notable cytotoxicity, although a slight decrease in viability was observed at 25 µM of all peptides except for NCR169C_17–38_W_10,20_/A, which had no effect on cell viability.

## 3. Discussion

The scarce resource and the side effect of antifungal compounds and the increasing number of resistant strains compel the search for new effective antifungals [21,22]. Antimicrobial peptides are promising candidates [23,24] as they have several beneficial characteristics i.e., broad-spectrum activity, low toxicity [25], moderate immunogenicity, good penetration capability, low propensity for development of resistance, distinct mode of actions and lack of cross resistance with the commonly used antifungals [26]. Natural antifungal peptides have been isolated from bacteria, fungi, plants, insects, amphibians, birds, and mammals and generally they possess a small size, overall positive charge, and amphipathic nature [27]. The majority of antifungal AMPs act via their interaction with the cell envelop provoking membrane permeabilization and leakage of cells. However, it is becoming increasingly apparent that peptides can have multifaceted mechanisms inhibiting various cell functions, such as nucleic acid and protein synthesis, and metabolism or cause production of reactive oxygen species and apoptosis [26]. Legume plants represent countless sources of symbiotic NCR peptides, many of which have broad ranges of antimicrobial and antifungal activities without notable toxicity on human cells [9]. Isolation of NCRs from root nodules is not feasible due to the small 1–3 mm size of nodules. Moreover, production of several hundreds of NCRs in the nodule with similar sizes and many with similar physicochemical properties would allow isolation of a mixture and not individual NCRs. Their production in heterologous expression systems is also challenging due to their strong antimicrobial activities. Thus, chemical synthesis of NCRs is the most straightforward way for their production, which, however, can relatively be costly for larger peptides.

In this study, the starting natural antifungal peptide for designing new, shorter drug candidates was the unusually long NCR335 peptide. The other peptide, NCR169 had no antifungal activity [14] but based on the high positive charge of the C-terminal half, we presumed that this sequence might have antifungal activity and was tested as a proof of concept. The antifungal activities of peptides were assessed against five *Candida* species and altogether 7 strains including intrinsically resistant ones. Our work demonstrated that truncated derivatives of NCR335 from both the N- and C-terminal halves as well as the C-terminal half of NCR169 have anticandidal activity.

All the NCR335N derivatives have a high positive charge (NCR335N_1–19_, NCR335N_1–15_ and NCR335N_7–21_: +5 NCR335N_16–29_: +4.1) but only NCR335N_7–21_ exhibited a broad spectrum activity, inhibiting the growth of all tested strains of *C. albicans, C. glabrata* and *C. tropicalis* but were ineffective against *C. auris* and *C. parapsilosis*. NCR335N_1–19_ was only effective against *C. albicans* ATCC 10231 and *C. parapsilosis* while NCR335N_1–15_ and NCR335N_16–29_ despite their ‘+’ net charge had no anticandidal activity in the tested concentration range. These results indicate that the ‘+’ net charge itself is not sufficient for the antifungal activity. Interestingly the predicted 3D structure of NCR335N_7–21_ peptide exhibiting the broadest spectrum appears to be alpha helical and different from the other NCR335N derivatives, indicating that the helical region might be important for the antifungal activity.

Of the C-terminal fragments of NCR335: NCR335C_1–33/13–33/17–33/17–27_ had a +6.8-6.9 net charge. NCR335C_1–33_ was effective against *C. albicans*, *C. glabrata* and *C. parapsilosis* while it did not affect the growth of *C. auris* and *C. tropicalis*. Its shorter derivative, NCR335C_17–33_ was even more active and effective against all strains including *C. tropicalis* but not against *C. auris*. NCR335C_13–33_ was only effective against *C. albicans* SC 5314, which together with the restricted efficacy of NCR335N_1–19_ against *C. albicans* ATCC 10231 indicates significant differences in the susceptibility of the studied strains of *C. albicans.* Truncating further the sequence of NCR335C_17–33_, resulting in NCR335C_17–27_ and the loss of activity suggests that the C-terminal sequence (C_18–33_) is indispensable for the antifungal action. The two 8 amino acid long peptides, NCR335C_1–8_ and NCR335C_9–16_ having +0.1 and +1.9 charge, respectively were inactive. The predicted folding of NCR335C_1–33_, NCR335C_17–27_ and NCR335C_13–33_ sequences indicates a helical segment at the N-terminus in the otherwise unordered structure. In contrast, the 3D structure of the most active NCR335C_17–33_ peptide revealed two short β strands but no helical region.

Testing C-terminal part of the NCR169 sequence has confirmed our assumption as NCR169C_17–38_ has indeed anticandidal activity and was effective against all *Candida* species and strains except for *C. parapsilosis.* There was some difference between the linear and oxidized forms as the latter was somewhat more effective on the *C. albicans* strains and significantly more effective against *C. glabrata*. The reason for the differential activity of the linear and oxidized forms can only be speculated, though it is known for NCR247 that its reduced and oxidized forms affect distinctly transcription and translation in bacteria [28,29]. Thus, likewise the cellular responses to linear and oxidized forms of NCR169C_17–38_ may be different. Tryptophan in antifungal peptides can play a critical role [18]. Replacement of W to A in NCR169C_17–38_ W_10,20_/A had only mild effect on the anticandidal activity except for the lost activity against *C. auris* indicating that tryptophan is essential in NCR169C_17–38_ against *C. auris* but not for the other *Candida* species. Cationic NCR peptides can provoke membrane permeabilization on a concentration dependent manner and by entering bacteria or fungi can have multiple intracellular targets [9]. NCR335, NCR247 and NCR192 were shown to provoke membrane permeabilization of *C. albicans* cells but at sublethal doses NCR247 was detected both in the fungal membrane and in the cytosol [14]. NCR044 was shown to bind to fungal cell wall and multiple membrane phospholipids, and by penetrating the membrane it accumulated in the cytoplasm and localized to the nuclear region [15]. Thus, most likely the antifungal, anticandidal NCRs exert multifaceted mechanisms like in bacteria where NCRs affect various cellular functions and provoke death of bacteria within minutes. Likewise, the active NCR peptide derivatives in this study caused not only growth inhibition but also killing of *Candida* cells. The yeast to hypha transition is critical to the pathogenesis of *C. albicans* [30]. Treatment of the dimorphic *C. albicans* and *C. tropicalis* with NCR335C_17–33_, NCR169C_17–38_ and NCR169C_17–38_ox resulted in the inhibition of filamentation and thus decreased pathogenesis of these species.

Biofilms are much more resistant to antimicrobials than planktonic cells. We show that all the active peptides inhibited biofilm formation of *C. albicans* and *C. tropicalis* either at the MIC or below, at ½ MIC and ¼ MIC.

Combined application of antifungal peptides with each other or with other antifungal drugs can be beneficial resulting in significant reduction of the MIC values compared to their MICs in single use. In a recent study combined action of two antifungal peptides from venom glands, ToAP2 and NDBP-5.7 with each other and with fluconazole or amphothericin B against *C. albicans* resulted in up to 4× reduced MICs compared to the compounds alone [31]. In our work, we assessed the interaction of NCR169C_17–38_ with NCR169C_17–38_ox and with NCR335C_17–33_ as well as the interaction of fluconazole with these peptides against *C. auris*. Interestingly synergistic interactions were detected between NCR169C_17–38_ and NCR169C_17–38_ox at 2× and 8× lower MICs, respectively, supporting the possibility that the linear and the oxidized form affect different cell functions. Synergism was also found between fluconazole and NCR169C_17–38_ox at 4× and 8× reduced MICs, respectively, and between NCR169C_17–38_ at 4× reduced MIC and NCR335C_17–33_ at 3.12 μM. This latter interaction is particularly interesting since NCR335C_17–33_ itself had no activity against *C. auris*.

The cytotoxicity of antifungal agents can largely limit their therapeutic potential. Testing the cytotoxicity of all the active NCR peptide derivatives up to 25 μM on human keratinocyte HaCaT cells showed no or only negligible toxicity and 25% reduction of cell viability was only observed at 25 μM. This cytotoxicity can be, however overcome with combined application of peptides with antifungal drugs necessitating much lower MICs of the peptides.

Our work thus demonstrates that the advantageous characteristics of these NCR peptide derivatives such as their broad-spectrum fungicidal activity, inhibition of the yeast to hypha transition and biofilm formation, their synergistic interactions with antifungals and their low toxicity on human cells make them promising anticandidal therapeutic drug candidates.

## 4. Materials and Methods

### 4.1. Peptide Synthesis

Peptides were synthesized according to the standard procedure of the solid-phase peptide synthesis (SPPS) by using an automatic peptide synthesizer (CEM Liberty Blue, Matthews, NC, USA) on TentaGel S RAM resin (the loading of the amino groups was 0.23 mmol/g). The applied chemistry utilized the Fmoc amino protecting group and diisopropylcarbodiimide/oxyma coupling with a fivefold excess of reagents. Removal of the fluorenyl-9-methoxycarbonyl group was carried out with 10% piperazine and 0.1 mol 1-hydroxy-benzotriazole dissolved in 10% ethanol and 90% DMF in 2 cycles (75 °C, 15 s and 90 °C, 50 s). After completion of the synthesis, the peptides were detached from the resin with a 95:5 (*v*/*v*) trifluoroacetic acid (TFA)/water mixture containing 3% (*w*/*v*) dithiothreitol and 3% (*w*/*v*) triisopropylsilane at room temperature for 3 h. The resin was removed by filtration and the peptides were precipitated by the addition of ice cold diethyl ether. Next, the precipitate was filtered, dissolved in water and lyophilized. The crude peptides were analyzed and purified by reverse-phase high-performance liquid chromatography (RP-HPLC). Peptides were purified using a C18 column; Perfectsil™ 100 ODS-3, 5 µ 20 × 250 mm, flow 4 mL/min for preparative separation and Perfectsil™ 100 ODS-3, 5 µ 4.6 × 250 mm, flow 1 mL/min for analytical investigations (MZ-Analysentechnik, Mainz, Germany) with a solvent system of (A) 0.1% (*v*/*v*) TFA in water and (B) 80% (*v*/*v*) acetonitrile and 0.1% TFA (*v*/*v*) in water at a flow rate of 4.0 mL min^−1^. The absorbance was detected at 220 nm. The appropriate fractions were pooled and lyophilized. Purity of the final products was characterized by analytical RP-HPLC at a flow rate of 1.0 mL min^−1^. The identity of the peptides was proved by ESI-MS spectrometry using Waters SQ detector (Milford, MA, USA). Peptide structures from amino acid sequences were predicted with the PEP-FOLD3 approach of Lamiable et al. available at https://bioserv.rpbs.univ-paris-diderot.fr/services/PEP-FOLD3/ [32,33,34].

### 4.2. Strains and Growth Conditions

All the *Candida* strains (Table 4) were grown overnight in YPD medium (1% pepton, 1% dextrose, 0.5% yeast extract) at 30 °C in water bath shaker prior to each experiments. The cells were harvested by centrifugation (5 min, 3000× *g*) washed twice with sterile distilled water and suspended in 5-fold diluted Difco Yeast Nitrogen Base *w*/*o* Amino Acids medium (Becton, Dickinson and Company, Sparks, MD, USA) supplemented with 1% dextrose, referred to as YNB medium in the text. Cells were counted in Bürker chamber and diluted to the proper concentration.

To induce morphological change of *C. albicans* and *C. tropicalis* YNB medium was changed for eight-fold diluted AIM-V+AlbuMAX (BSA) medium (Gibco, Thermo Fisher Scientific, Dublin, Ireland) referred to as 1/8AIM in the text.

### 4.3. Antifungal Activity Assays

The growth inhibition of the peptides listed in Table 1 was tested in 96-well microtiter plates on *Candida* strains. The minimal inhibitory concentration (MIC) was determined with the micro-dilution method by adding 5 µL serially two-fold-diluted peptide solution to 95 µL of cell suspension (4 × 10^4^ cell/mL) in YNB medium. After 48 h of incubation at 30 °C the optical density of the cultures was measured at 620 nm in SPECTROstar Nano plate reader (BMG LabTech, Offenburg, Germany). Minimal inhibitory concentration was defined as growth inhibition ≥90% compared to 100% growth of the untreated control. The experiments were carried out in three biological repeats always in triplicates.

The same initial experimental setting was used to determine the fungicide effect of the peptides. Five µL samples were taken from the cultures after 24 h and were added to 95 µL sterile distilled water, and diluted to 10- and 100-fold (indicated as 10^4^, 10^3^ and 10^2^ based on the cell number of the control culture in Figure A2). Five µL from each dilution were placed on solid YPD medium and the growth of the strains was detected after 48 h incubation at 30 °C.

### 4.4. Biofilm Formation Assay of C. albicans and C. tropicalis Strains

A total of 95 µL of cell suspensions at 4 × 10^4^ cell/mL in 1/8AIM medium were added into the wells of microplates and supplemented with 5 µL of two-fold dilution series of the peptides or 5 µL of medium as control. Plates were incubated at 37 °C in 5 % CO_2_ level for 72 h and the formed biofilms were washed twice with phosphate buffered saline (PBS) to remove the slightly attached cells. The viability of the biofilm-embedded cells was measured with the XTT reduction assay. XTT was solved in PBS at 0.5 mg/mL concentration and supplemented with 1 µM menadion. After adding 100 µL XTT solution to each well the plates were incubated for 2 h at 37 °C in dark. Subsequently 80 µL of each supernatant was transferred to new 96-well plates and the absorbance was measured at 490 nm using SPECTROstar Nano plate reader (BMG LabTech, Offenburg, Germany). The experiments were carried in 5 biological replicates in duplicates.

### 4.5. Morphological Analysis of C. albicans and C. tropicalis Cells

Bright-field microscopy. The morphology of *C. albicans* ATCC 10231 and *C. tropicalis* cells was examined after 48 h incubation with the selected peptides (NCR335C_17–33_ and NCR169C_17–38_) by Zeiss Axio Observer inverted microscope (Carl Zeiss AG, Jena, Germany). The concentration of the NCR335C_17–33_ was 1.56 and 3.12 µM, NCR169C_17–38_ 1.56 or 3.12 µM while NCR169C_17–38ox_ 0.78 or 3.12 µM, the initial cell concentration was set to 4 × 10^4^ cell/mL. Cells cultivated without peptides were used as control.

Scanning electron microscopy. Polyethylene terephtalate, glycol-modified (PET-G) cover slips (Sarstedt, Nümbrecht, Germany) were placed into the wells of 12-well microtiter plate. 600 µL of *C. albicans* ATCC 10231 and *C. tropicalis* suspensions at 4 × 10^4^ cells/mL were loaded into the wells and treated with the selected peptides for 72 h at 37 °C in 5% CO_2_ level the cultivation medium was removed and the samples were washed with PBS. Cells were fixed with 2.5% glutaraldehyde in PBS for 2 h at room temperature. After the fixation, *C. tropicalis* cells were filtered on poly-L-lysine-coated polycarbonate filters. Then both *C. albicans* and *C. tropicalis* samples were dehydrated in aqueous solutions of increasing ethanol concentrations, critical point dried, covered with 15 nm gold by a Quorum Q150T ES sputter (Quorum, Laughton, UK) and observed in a JEOL JSM-7100F/LV scanning electron microscope (Jeol Ltd., Tokyo, Japan).

### 4.6. Combined Treatment of C. auris with Fluconazole and Selected Peptides

The effects of the different combinations of fluconazole and NCR335C_17–33_, NCR169C_17–38_ or NCR169C_17–38_ox were determined by standard checkerboard titration method [35]. The fluconazole was tested in a concentration range from 6.25 to 75 µM, NCR169C_17–38_ and NCR169C_17–38_ox from 0.78 to 12.50 µM, NCR335C_17–33_ from 1.56 to 25 µM. The initial cell concentration in each well was 4 × 10^4^ cell/mL. After the incubation for 72 h at 30 °C, the optical density of the cultures was detected at 620 nm in SPECTROstar Nano plate reader (BMG LabTech, Offenburg, Germany). The inhibitory concentrations were determined for each compound alone and in combinations. The experiments were carried out at least three times. The effect of combinations was evaluated by fractional inhibitory concentration (FIC) index. FIC = FIC_A_ + FIC_B_. FIC_A_ = (MIC_A_ in combination)/(MIC_A_ alone); FIC_B_ = (MIC_B_ in combination)/(MIC_B_ alone)

### 4.7. Assessment of the Viability of Human Keratinocytes

The viability of human keratinocytes (HaCaT) was detected after peptide-treatments and compared to the untreated control. Briefly, HaCaT cells were seeded into 96-well micro-plates (10,000 cells/well) then cultured in a 37 °C incubator at 5% CO_2_ in 95% humidity. On the following day, the cells were treated with increasing concentrations of NCR335 and NCR169C derivatives. After 48 h treatments, HaCaT cells were washed with PBS (phosphate buffered saline) and incubated for an hour at 37 °C with MTT reagent (Sigma-Aldrich, St. Louis, MO, USA) at 0.5 mg/mL concentration diluted in the culture medium. Formazan crystals were solubilized in DMSO (Sigma-Aldrich, St. Louis, MO, USA) and the absorption was measured at 570 nm in SPECTROstar Nano plate reader (BMG LabTech, Offenburg, Germany). The experiments were performed at least three times using four independent biological replicates.

### 4.8. Statistical Analysis

Data represent the mean ± standard deviation (SD) calculated from at least three independent experiments. Statistical analysis was performed by using GraphPad Prism v6.07 (GraphPad Software, Inc., La Jolla, CA, USA). The unpaired *t* test was used and results were considered statistically significant when *p* ≤ 0.05.

## 5. Conclusions

Natural NCR peptides represent an exceptionally rich source of antimicrobial activities. Here we provided evidence on the example of two peptides, NCR335 and NCR169, that their synthetic truncated derivatives can maintain and even gain anticandidal activities. The anticandidal peptides are able to inhibit the yeast to hypha morphological switch and biofilm formation of *C. albicans* and *C. tropicalis* at low concentrations. Importantly, NCR169C_17–38_ proved to be effective against the multidrug resistant species, *C. auris*. Synergistic interactions of the peptides with each other or with antifungal drugs make them even more attractive and increase their therapeutic potential. The few known examples of antifungal peptides and the need for novel antibacterial and antifungal agents urge further exploitation of the NCRs’ activities and advantageous properties.

## Figures and Tables

**Table 1:** Physicochemical properties of NCR peptides synthetized with C-terminal amidation.

Peptide	Sequence of the Peptide	No. AA	pI	Net Charge
NCR335N_1–19_	RLNTTFRPLNFKMLRFWGQ	19	14	+5
NCR335N_1–15_	RLNTTFRPLNFKMLR	15	14	+5
NCR335N_7–21_	RPLNFKMLRFWGQNR	15	14	+5
NCR335N_16–29_	FWGQNRNIMKHRGQ	14	14	+4.1
NCR335C_1–33_	HFSLILSDCKTNKDCPKLRRANVRCRKSYCVPI	33	10.37	+6.8
NCR335C_13–33_	KDCPKLRRANVRCRKSYCVPI	21	10.91	+6.8
NCR335C_17–33_	KLRRANVRCRKSYCVPI	17	11.73	+6.9
NCR335C_17–27_	KLRRANVRCRK	11	12.59	+6.9
NCR335C_1–8_	HFSLILSD	8	7.57	+0.1
NCR335C_9–16_	CKTNKDCP	8	9.23	+1.9
NCR335C_1–16_	HFSLILSDCKTNKDCP	16	8.07	+1.0
NCR169C_17–38_	KSKKPLFKIWKCVENVCVLWYK	22	10.48	+5.9
NCR169C_17–38_ox	KSKKPLFKIWKĈVENVĈVLWYK	22	11.01	+6
NCR169C_17–38_W_10,20_/A	KSKKPLFKIAKCVENVCVLAYK	22	10.48	+6

Ĉ indicates two cysteines joined by disulphide bond. Alanin residues replacing W_10_ and W_20_ are underlined.

**Table 2:** Minimal inhibitory concentration (µM) of the NCR peptide fragments against *Candida* strains.

Peptide	Ca ATCC 10231	Ca SC 5314	Ca SZMC 1458	Cau 0381	Cg CBS 138	Cp CBS 604	Ct CBS 94
NCR335N_1–19_	25	-	-	-	-	12.50	-
NCR335N_1–15_	-	-	-	-	-	-	-
NCR335N_7–21_	25	25	25	-	25	-	6.25
NCR335N_16–29_	-	-	-	-	-	-	-
NCR335C_1–33_	25	12.50	12.50	-	25	25	-
NCR335C_13–33_	-	12.50	-	-	-	-	-
NCR335C_17–33_	12.50	12.50	12.50	-	25	12.50	3.12
NCR335C_17–27_	-	-	-	-	-	-	-
NCR335C_1–8_	-	-	-	-	-	-	-
NCR335C_9–16_	-	-	-	-	-	-	-
NCR335C_1–16_	-	-	-	-	-	-	-
NCR169C_17–38_	25	12.50	12.50	6.25	25	-	3.12
NCR169C_17–38_ ox	6.25	6.25	6.25	12.50	6.25	-	3.12
NCR169C_17–38_W_10,20_/A	25	12.50	6.25	-	12.50	-	6.25
Fluconazole	200	12.50	1.56	25	200	6.25	100
Amphotericin B	3.12	3.12	3.12	6.25	1.56	3.12	6.25

-: no growth inhibition was observed. Ca: *Candida albicans*, Cau: *Candida auris*, Cg: *Candida glabrata*, Cp: *Candida parapsilosis*, Ct: *Candida tropicalis*.

**Table 3:** Combined activity of NCR peptide derivatives and fluconazole.

Drug A	Drug B	FIC_A_ (µM)	FIC_B_ (µM)	FIC	Action
Fluconazole	NCR335C_17–33_	1 (25)	0.125 * (1.56)	1.125 *	Indifferent
	NCR169C_17–38_	0.5 (12.50)	0.125 (0.78)	0.625	Additive
	NCR169C_17–38_ox	0.25 (6.25)	0.25 (1.56)	0.5	Synergism
NCR169C_17–38_	NCR335C_17–33_	0.125 (1.56)	0.25 * (3.12)	0.375 *	Synergism
	NCR169C_17–38_ox	0.25 (3.12)	0.25 (1.56)	0.5	Synergism

Fractional inhibitory concentration (FIC) index values: ≤0.5: synergism; 0.5 < FIC ≤ 1.0: additive; 1.0 < FIC ≤ 2.0: indifferent; >2: antagonism. (µM) corresponds to the concentration of drug A and Drug B for the indicated action. * represents a fictitious FIC value which could be lower as NCR335C_17–33_ was inactive against *C. auris* at 25 µM nevertheless 25 µM was used as MIC for the calculation of FIC.

**Table 4:** List of the tested strains.

*Species*	*Strain Number*
*Candida albicans*	ATCC 10231
*Candida albicans*	SC 5314
*Candida albicans*	SZMC 1458
*Candida auris*	0381
*Candida glabrata*	CBS 138
*Candida parapsilosis*	CBS 604
*Candida tropicalis*	CBS 94

ATCC: American Type Culture Collection; CBS: Centraalbureau voor Schimmelcultures; SC: Squibb Institute for Medical Research, New Brunswick, NJ, US; SZMC: Szeged Microbiological Collection.

## Data Availability

The supporting data of these findings are available on request from the corresponding author, [I.P.].
